# Systematic Identification of Candidate Substrates of the UCH Family of Deubiquitinases

**DOI:** 10.3390/ijms27209217

**Published:** 2026-10-17

**Authors:** Juanma Ramirez, Gorka Prieto, Erik P. Barrio, Ainhoa Atxa-Espiga, Natalia Presa, Olatz Arteaga-Cabeza, Ugo Mayor, Nerea Osinalde

**Affiliations:** 1 Department of Biochemistry and Molecular Biology, Faculty of Science and Technology, University of the Basque Country (EHU), 48940 Leioa, Spain; juanmanuel.ramirez@ehu.eus (J.R.); ainhoa.atxa@ehu.eus (A.A.-E.); natalia.presa@ehu.eus (N.P.); 2 Department of Communications Engineering, University of the Basque Country (EHU), 48013 Bilbao, Spain; gorka.prieto@ehu.eus; 3 Department of Biochemistry and Molecular Biology, Faculty of Pharmacy, University of the Basque Country (EHU), 01006 Vitoria-Gasteiz, Spain; erik.paco@ehu.eus (E.P.B.); olatz.arteaga@ehu.eus (O.A.-C.); 4 Ikerbasque, Basque Foundation for Science, 48013 Bilbao, Spain

**Keywords:** deubiquitinase, candidate substrate, UCH, bioUb, diGly site, LFQ-MS

## Abstract

Deubiquitinases (DUBs) of the ubiquitin C-terminal hydrolase (UCH) family play critical roles in regulating ubiquitin-dependent signaling, yet their substrate specificity and functional organization remain incompletely defined. Here, we systematically characterized the ubiquitination landscape associated with UCH family members (UCHL1, UCHL3, UCHL5, and BAP1) using a quantitative proteomics workflow combining siRNA-mediated DUB silencing, bioUb-based enrichment of ubiquitinated proteins, and label-free mass spectrometry. Proteins exhibiting increased ubiquitination following DUB depletion were identified as candidate substrates. Our analysis revealed that UCH family members regulate largely distinct subsets of ubiquitinated proteins, with limited overlap in their candidate profiles despite sharing conserved catalytic domains. In parallel, site-resolved ubiquitinomics identified 851 ubiquitination sites, including 106 not previously reported, expanding the current landscape of experimentally validated ubiquitin-modified lysines. Functionally, substrate networks link individual UCH enzymes to distinct yet partially overlapping cellular processes, including hypoxia signaling, RNA metabolism, vesicle trafficking, apoptosis, and chromatin regulation. Notably, known substrates such as HIF-1α validated the approach, while recurrent detection of key regulators, including MCL1, pointed to convergence at the level of apoptosis control. Together, these findings provide a resource for exploring the distinct and potentially overlapping roles of UCH deubiquitinases within cellular ubiquitin-regulatory networks.

## 1. Introduction

Ubiquitination is a universal post-translational modification that affects the vast majority of cellular proteins. Initially described in the 1970s as a degradation signal for the proteasome [1], it is now recognized as a highly versatile regulatory mechanism. An expanding array of non-degradative ubiquitin signals has been identified, each fine-tuning essential cellular processes including DNA repair, autophagy, transcriptional regulation, and receptor internalization [2]. Ubiquitination involves the covalent attachment of ubiquitin to protein substrates or previously conjugated ubiquitin allowing the formation of polyubiquitin chains with diverse and often complex topologies. Ubiquitin itself can be modified by ubiquitin-like proteins such as SUMO (small ubiquitin-like modifier) or NEDD8 (Neural Precursor Cell Expressed, Developmentally Down-Regulated 8), as well as by phosphorylation or acetylation. Together, these modifications form a sophisticated “ubiquitin code” that instructs specific downstream outcomes [3,4].

Ubiquitination involves the sequential action of E1 ubiquitin-activating enzymes, E2 ubiquitin-conjugating enzymes, and E3 ubiquitin ligases, which together mediate the transfer of ubiquitin to substrate proteins [5]. Like most post-translational modifications, ubiquitination is reversible. Deubiquitinating enzymes (DUBs) cleave peptide and isopeptide bonds between ubiquitin and substrates, remodel ubiquitin chains, and process ubiquitin precursors to maintain free ubiquitin pools that are essential for cellular homeostasis [4]. Consequently, DUBs influence virtually all biological processes [6], and dysregulated DUB activity contributes to numerous human diseases, including cancer and neurodegeneration [7,8].

The human genome encodes nearly one hundred DUBs, grouped in seven major families, including six cysteine protease families— (i) ubiquitin-specific proteases (USPs), (ii) ubiquitin C-terminal hydrolases (UCHs), (iii) ovarian tumor proteases (OTUs), (iv) Machado–Josephin domain-containing proteases (MJDs), (v) MIU-containing novel DUBs (MINDYs), and (vi) zinc finger-containing ubiquitin peptidase 1 (ZUP1)—and the zinc-dependent metalloprotease family JAMM (JAB1/MPN/MOV34). Despite their diversity, these enzymes share a conserved catalytic cleft, making them attractive therapeutic targets. However, efforts to develop selective DUB modulators have been hindered by an incomplete understanding of DUB biology, particularly the specific substrates each enzyme acts upon. Although recent work has made important progress [9,10], substrate identification remains a major challenge.

Within the DUB landscape, the UCH family of cysteine proteases remains one of the least characterized. Its four members—UCHL1, UCHL3, UCHL5/UCH37, and BAP1—share a conserved 230-amino-acid catalytic UCH domain and an unstructured loop region that restricts access to the active site. The length and flexibility of this loop play central roles in determining substrate specificity and catalytic efficiency [11]. UCHL1, the smallest UCH family member, is highly abundant in brain tissue, where it accounts for up to 5% of soluble protein and exhibits both ligase and hydrolase activities [12,13]. UCHL1 and its closest homolog UCHL3 cleave the C-terminal glycine of ubiquitin or NEDD8 but do not hydrolyze diubiquitin or longer polyubiquitin chains [11,14,15]. In contrast, UCHL5 and BAP1 (BRCA1-associated protein 1) contain an additional C-terminal UCH37-like domain (ULD) that enables them to process K48-linked polyubiquitin chains in cooperation with specific cofactors [16,17]. UCHL5 associates with the 19S proteasome, where it both promotes substrate degradation by cleaving branched K48 chains [18] and can limit turnover by trimming chains on weakly ubiquitinated substrates [19]. BAP1, the largest family member, is a nuclear tumor suppressor whose deubiquitinase activity regulates numerous key cellular pathways [20].

Through these diverse biochemical activities, UCH enzymes modulate a wide range of signaling pathways, including nucleosome remodeling [17], synaptic plasticity [21], DNA damage repair [22], and processes underlying oncogenesis, cell invasion, and migration [23,24,25]. Consequently, UCH family dysregulation is implicated in a broad spectrum of human diseases, spanning nearly all cancer types [23,26,27,28], multiple neurodevelopmental disorders [29], and metabolic diseases such as diabetes [30]. These roles have established UCH enzymes, and their protein interactors and substrates, as promising therapeutic targets [31,32,33,34]. However, despite their functional importance, the direct substrates of most UCH enzymes remain largely uncharacterized.

The limited knowledge regarding UCH substrates reflects a broader challenge in deubiquitinase biology. Unlike many kinases or proteases, DUBs often recognize their substrates through context-dependent interactions involving adaptor proteins, subcellular localization, protein complexes, or specific ubiquitin chain architectures. As a result, direct substrate identification remains considerably more difficult than the characterization of catalytic activity itself. Although several substrates have been reported for individual UCH family members, including HIF-1α for UCHL1 [35] and H2A for BAP1 [36], a systematic comparison of candidate substrates across the entire UCH family has not been performed. This lack of comprehensive substrate maps limits our understanding of both the shared and family-member-specific functions of UCH deubiquitinases.

Proteomics provides a powerful and unbiased strategy to identify proteins whose ubiquitination status changes following DUB perturbation. We previously demonstrated that quantitative analysis of ubiquitinated proteins accumulating after DUB depletion enables systematic identification of candidate DUB substrates [37]. Here, we extend this strategy to the UCH family and systematically investigate the consequences of UCHL1, UCHL3, UCHL5, and BAP1 depletion on the cellular ubiquitin landscape. By integrating BioUb-mediated enrichment of ubiquitinated proteins with site-resolved ubiquitinomics, we sought to define candidate substrate repertoires and ubiquitination sites for each UCH family member and to compare their substrate profiles, providing a framework to investigate both shared and family-member-specific functions within this understudied DUB family.

## 2. Results

### 2.1. Proteomics Workflow to Identify Candidate Substrates of UCH Family DUBs

**Figure 1:**
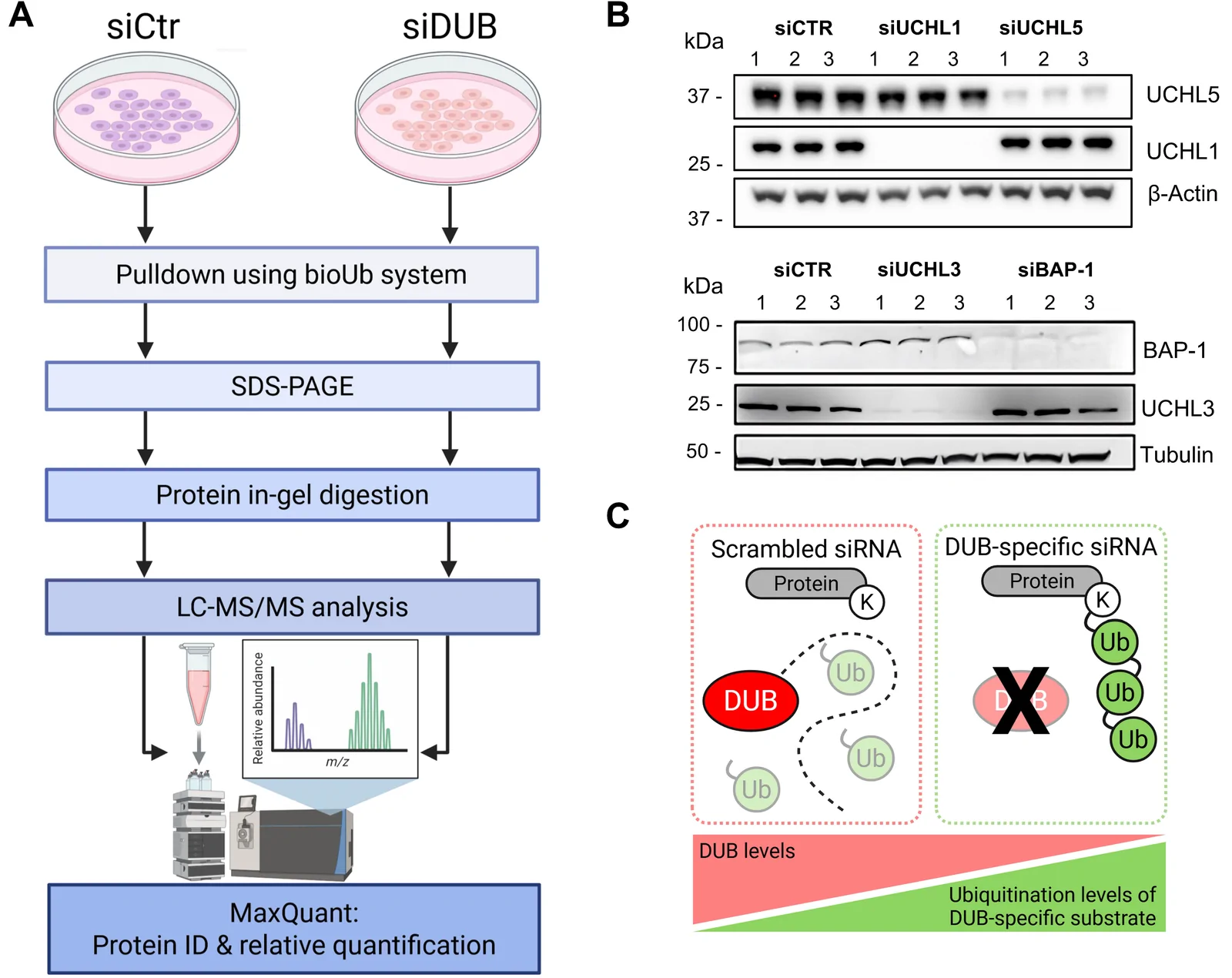
Strategy to uncover candidate substrates of UCH deubiquitinases. (**A**) Schematic representation of the workflow to detect putative substrates of UCH family members. In brief, ubiquitinated proteins were enriched using the bioUb system from cells transfected with either a scrambled control siRNA or siRNAs targeting UCHL1, UCHL3, UCHL5, or BAP1. Following SDS-PAGE separation and in-gel tryptic digestion of the enriched proteins, the resulting peptides were analyzed by label-free quantitative mass spectrometry. Each condition was analyzed in triplicate. (**B**) Prior to MS analysis, the silencing efficiency of each of the four UCH family members was verified by Western blotting. Tubulin and beta-actin were used as a loading control. (**C**) Silencing a specific deubiquitinase increases the ubiquitination of its candidate substrates.

The primary objective of this study was to identify candidate substrates of the UCH family of DUBs. To this end, we implemented the workflow outlined in Figure 1. As previously described [37], HEK293 cells were first transfected with siRNAs targeting each UCH member individually (Figure 1A). Cells were subsequently transfected with the bioUb construct [38], which encodes a precursor polypeptide composed of six biotinylatable ubiquitin molecules fused to the *E. coli* biotin ligase BirA. Once expressed, the precursor is processed by endogenous DUBs, releasing BirA and enabling it to biotinylate the 16-amino-acid biotinylation motif positioned at the N-terminus of each ubiquitin unit. The resulting biotinylated ubiquitin behaves similarly to endogenous ubiquitin and is readily incorporated into cellular substrates by the ubiquitination machinery [38].

Cells were then lysed, and efficient knockdown of each UCH DUB was confirmed by Western blotting (Figure 1B). Additionally, we assessed whether depletion of individual UCH family members altered the overall ubiquitin pool, with particular attention to UCHL3 and UCHL5, which have been implicated in ubiquitin processing and homeostasis [15,18]. We observed no apparent changes in overall ubiquitination or free ubiquitin following UCH family member silencing (Figure S1), suggesting that the changes detected by bioUb-based proteomics are unlikely to reflect a substantial alteration in global ubiquitin availability.

Next, ubiquitinated proteins were enriched through streptavidin affinity purification. To eliminate avidin monomer and dimer contamination, the enriched material was fractionated by SDS-PAGE. Proteins within distinct gel slices were then subjected to in-gel reduction, alkylation, and trypsin digestion, as described previously [39]. The resulting peptides were analyzed by label-free quantitative (LFQ) mass spectrometry, enabling the identification of proteins whose ubiquitination levels increased upon DUB silencing (Figure 1B). Such increases are expected for putative substrates, as loss of the cognate DUB should lead to an accumulation of ubiquitinated forms (Figure 1C).

LFQ-MS analysis was performed with enriched ubiquitinated proteomes. This quantitative approach allowed us to compare ubiquitination levels between DUB-depleted and control samples and thereby identify proteins whose ubiquitination increased upon loss of each UCH family member, revealing candidate substrates for these deubiquitinases.

### 2.2. Large-Scale In Vivo Detection of UCH Family Substrates

**Figure 2:**
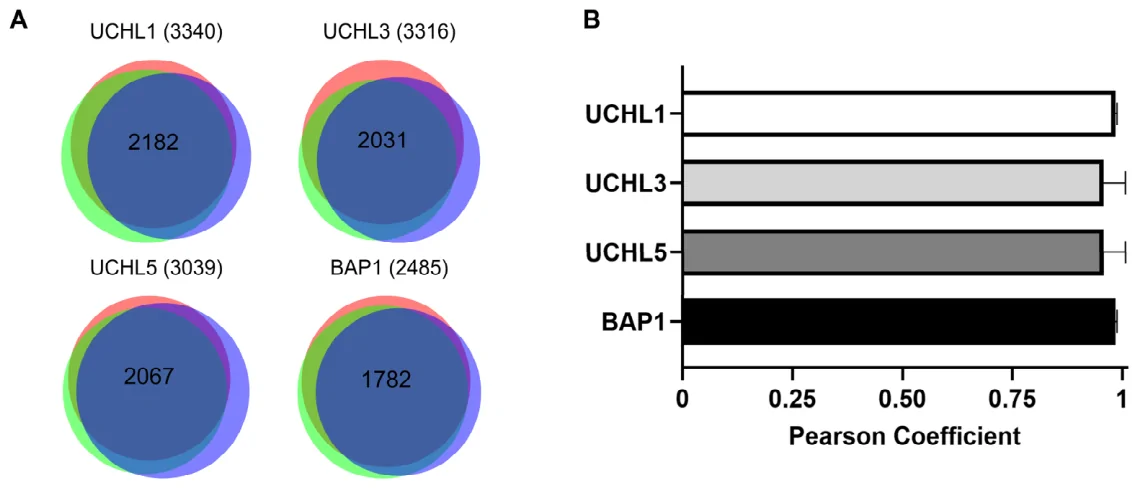
Reproducibility of identification and quantification of ubiquitinated proteins. (**A**) Venn diagram showing the overlap of proteins identified across four experimental conditions. The three colors represent the three biological replicates for each condition. The total number of proteins identified in each condition is indicated in parentheses, while the central intersection represents the number of proteins consistently detected in all three biological replicates of each condition. (**B**) Pearson correlation coefficients (median value) among the three biological replicates for each DUB-depleted experimental condition.

To evaluate the robustness and reliability of the ubiquitin-enriched proteomic datasets, we performed a comprehensive quality control analysis across all samples and experimental replicates. Protein identification was consistent across biological replicates. Overlap analysis showed that 71–89% of proteins were detected in at least two out of three replicates per condition, while 61–80% of proteins were consistently identified across all three replicates (Figure 2A). To complement this assessment, we examined the similarity of LFQ intensity profiles by calculating pairwise Pearson correlation coefficients across all samples within each experimental set. The biological replicates of all four UCH knockdown experiments displayed consistently high correlations, ranging from 0.974 to 0.987 (Figure 2B and Figure S2). As expected, correlations between knockdown and control samples were slightly lower (minimum values between 0.936 and 0.969) reflecting the biological differences introduced by DUB silencing (Figure S2). Together, the consistent replicate overlap and high inter-replicate correlations confirm the technical robustness of the dataset and support its suitability for confident identification of putative UCH family DUB substrates.

**Figure 3:**
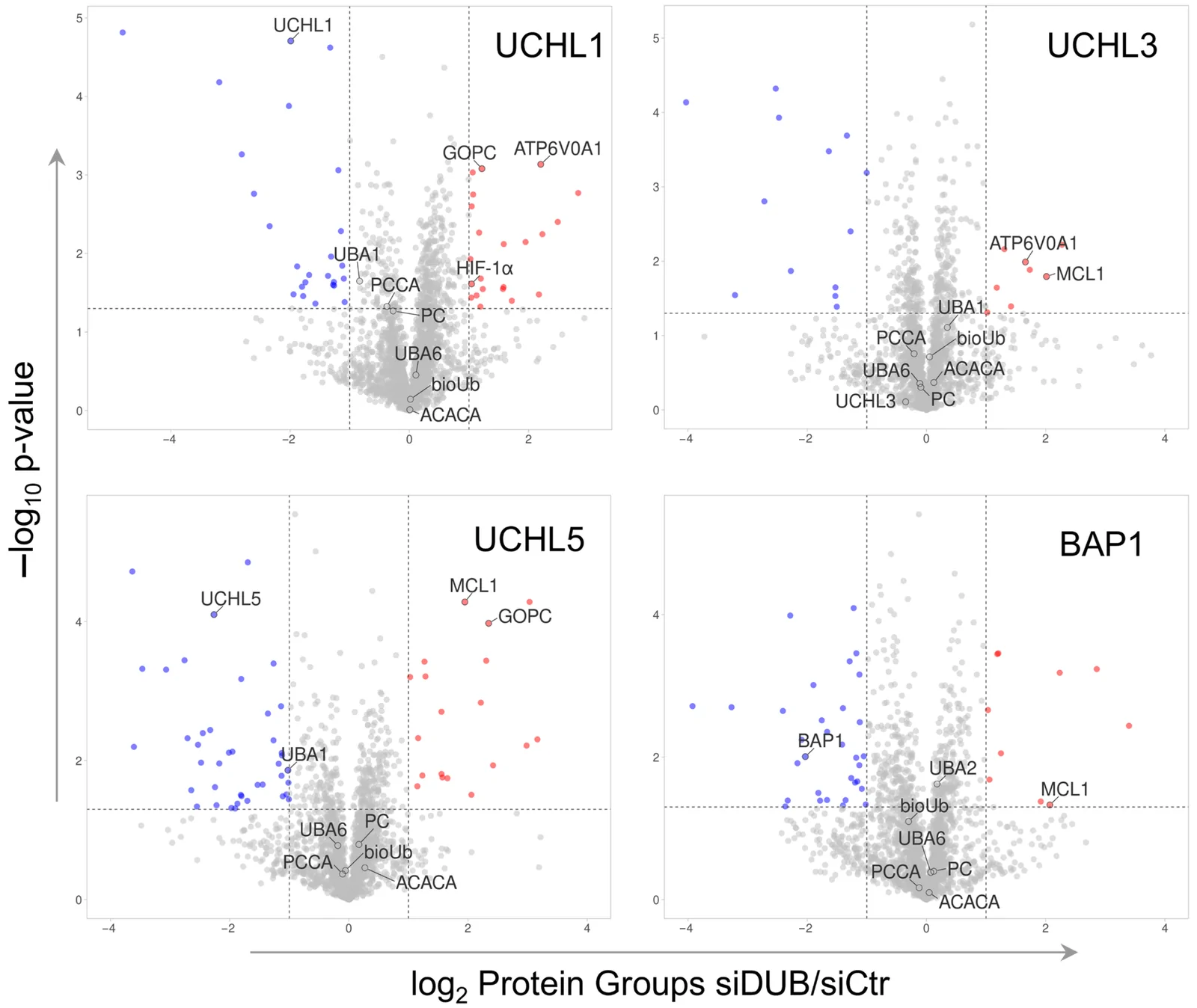
Identification of putative substrates of UCH family members. Volcano plots show differentially ubiquitinated proteins upon UCHL1, UCHL3, UCHL5 and BAP1 silencing, relative to control samples. The abundance of each protein in the enriched fraction was estimated from its label-free quantification (LFQ) intensity. The LFQ siRNA/control ratios (log_2_ scale) and the *t*-test *p*-values (−log_10_ scale) are displayed on the x- and y-axes, respectively. The threshold for statistical significance (*p*-value < 0.05) is indicated with a horizontal grey line, while vertical grey lines depict a two-fold increase or decrease in protein abundance in the ubiquitin-enriched fraction upon DUB silencing. Proteins with a 2-fold increase or reduction in their ubiquitination levels upon DUB silencing are shown in red and blue, respectively. Grey dots represent proteins that do not meet the criteria for statistically significant regulation.

To identify proteins whose ubiquitination status was affected by loss of each UCH family DUB, we compared LFQ intensities of ubiquitinated proteins between knockdown and control samples (Tables S1–S4) and visualized the results using volcano plots (Figure 3). As a first internal control, we checked for the specificity and robustness of the bioUb enrichment strategy, and examined proteins expected to remain unchanged across conditions. The bioUb precursor itself, as well as ubiquitin-activating enzymes, such as UBA1 and UBA6, which are likely to be isolated as bioUb carriers, consistently displayed ratios close to 1 between siDUB and siCTR samples, confirming comparable labeling and ubiquitination capacity across conditions (Figure 3; Tables S1–S4, highlighted in yellow and orange). In addition, the abundance of endogenously biotinylated carboxylases (ACACA, PC, and PCCA) remained constant across conditions (Figure 3; Tables S1–S4, highlighted in green), supporting comparable pull-down efficiency and sample input across conditions. Although we did not anticipate that the silenced DUBs themselves would be ubiquitinated, their detection in the bioUb pulldown suggested otherwise (Figure 3; Tables S1–S4).

As shown in Figure 3, silencing of each UCH produced a distinct pattern of ubiquitination changes, with defined subsets of proteins exhibiting increased (red dots) or decreased (blue dots) ubiquitination relative to control. Proteins with elevated ubiquitination, reflecting the accumulation of ubiquitinated species upon loss of the corresponding DUB, represent the most plausible candidate substrates of the given DUB. Conversely, reduced ubiquitination likely reflects indirect or compensatory adjustments within the ubiquitin system. These bidirectional changes illustrate that depletion of each UCH member reshapes the cellular ubiquitin landscape in a unique manner, providing a mechanistic basis for the functional specificity observed among UCH family members.

Having established the overall ubiquitination profiles, we next focused on proteins that displayed increased ubiquitination upon DUB depletion (log_2_ [siDUB/siCtr] > 1 and *p* < 0.05), as these represent the most compelling candidates for UCH DUB substrates (Table 1). It is worth noting that we identified hypoxia-inducible factor 1α (HIF-1α), a previously reported UCHL1 substrate, among our candidates [35]. This result underscores the reliability of our approach and supports confidence in newly identified candidate targets.

Each UCH family member regulates a largely non-overlapping substrate spectrum, while still converging on specific molecular nodes (Table 1). UCHL1 displayed the broadest candidate substrate repertoire, spanning proteins involved in diverse cellular processes, with prominent representation of regulators of cell proliferation and transcription, including CCND2, CDKN1A, MTA3, CREB3L1, SMG1, CHD8, FYN, and EP300, alongside vesicle-trafficking factors such as STXBP2, RAB10, and the V-ATPase subunit ATP6V0A1, which was also identified among the candidate substrates of UCHL3. Putative UCHL3 substrates included, among others, actin-remodeling and signaling components (WASF2, NUMB, PIK3C3), nuclear transport factors (NUP50), and transcriptional regulators (GABPA, MRFAP1). Notably, MCL1 was strongly enriched following UCHL3 depletion, which also emerged as a putative substrate of UCHL5 and BAP1, suggesting multi-DUB regulation of an MCL1-centered survival module. The candidate substrates identified upon UCHL5 depletion encompassed UCHL5 proteins involved in diverse cellular functions, including signaling (HRAS), ER-associated quality control (VIMP), vesicle trafficking (GOPC), cytoskeletal and chromatin regulation (EML4, TRRAP, RAD54L2), RNA processing (DDX10), and translation (EIF3J). Finally, bioUb pull-downs from BAP1-depleted cells identified putative substrates involved in signaling and trafficking (MET, TNFRSF10D), RNA processing (THOC5), proteolysis (CAPN7), ubiquitin-pathway regulation (UBA1), and translation (EIF3F, SPG20).

Overall, overlap between DUB datasets was limited, with most candidate substrates unique to individual UCH family members. MCL1 was the only protein identified following depletion of three DUBs (UCHL3, UCHL5, and BAP1), whereas ATP6V0A1 and GOPC were shared between the UCHL1/UCHL3 and UCHL1/UCHL5 datasets, respectively. Collectively, these findings suggest that although individual UCH family DUBs are associated with largely distinct substrate repertoires, they converge on a limited set of proteins involved in membrane trafficking, transcriptional regulation, and apoptosis.

To facilitate independent assessment of candidate selection criteria, all datasets generated in this study have been incorporated into DUBase, our publicly accessible repository of DUB-substrate discovery experiments [37]. Users can interactively explore volcano plots and modify statistical and fold-change thresholds according to their desired level of stringency. This will enable researchers to evaluate candidate substrates under more permissive or more conservative filtering strategies and to compare the resulting candidate lists directly.

### 2.3. Uncovering Novel Ubiquitination Sites

In our experimental workflow, ubiquitinated proteins were enriched at the protein level. Subsequent digestion and LC–MS/MS analysis allowed us to identify unmodified peptides for protein assignment while simultaneously detecting diGly-modified peptides that reported site-specific ubiquitination events.

**Figure 4:**
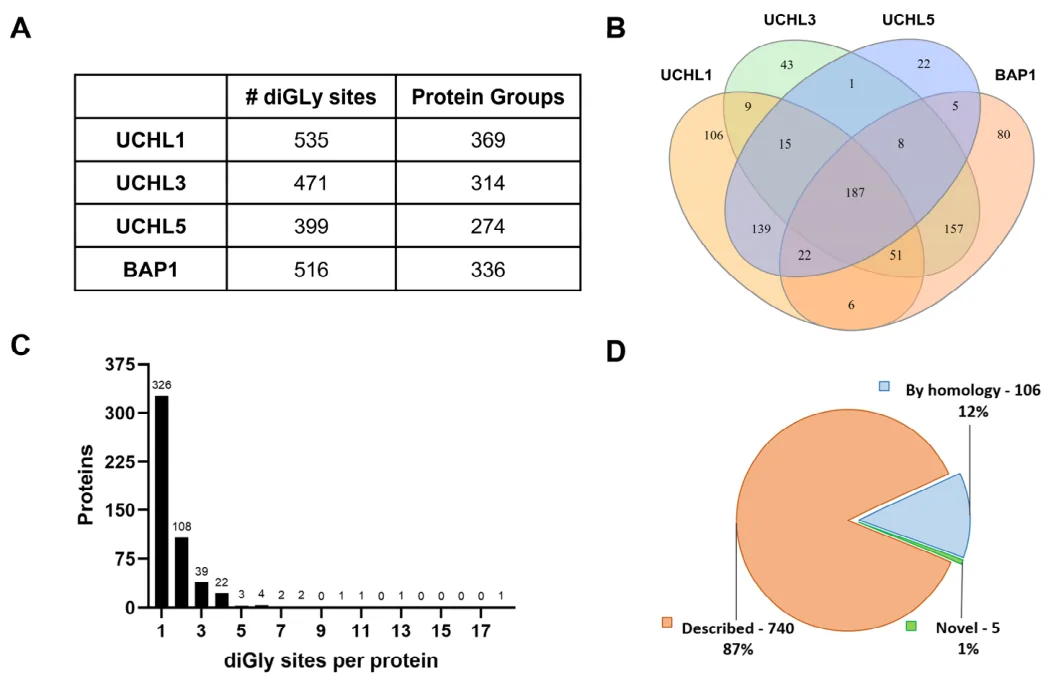
Overview of diGly site identification across DUB-silencing experiments. (**A**) Summary table reporting the total number of ubiquitination sites (#diGly sites) and the corresponding number of distinct protein groups detected in each experimental condition. (**B**) Venn diagram showing the overlap of diGly sites identified across the four DUB knockdown datasets, illustrating shared and unique ubiquitination sites. (**C**) Distribution of the number of diGly sites per protein, indicating that most proteins carry a single ubiquitination site, while a smaller subset contains multiple sites. (**D**) Annotation status of identified diGly ubiquitination sites in the PhosphoSitePlus database. Sites are categorized as previously reported in humans, reported in other species, or newly described in this study.

Across the four experimental conditions we identified between 499 and 535 ubiquitination sites, corresponding to 269–357 distinct protein groups (Figure 4A, Table S5). The Venn diagram (Figure 4B) illustrates a considerable overlap in ubiquitination sites detected among these datasets, with 187 sites shared across all conditions. Notably, each dataset also contained unique sites, reflecting DUB-specific ubiquitination sites. For most proteins, only one diGly site was detected, whereas a smaller subset harbored multiple ubiquitination sites (Figure 4C).

In total, across all conditions, we detected 851 unique diGly sites mapping to 510 distinct protein groups. To evaluate the novelty of the ubiquitination events detected in our study, we compared them with the ubiquitination annotations available in the PhosphoSitePlus database (Table S6) [40]. This analysis revealed that 106 of the identified sites are not present in the database and therefore represent previously unreported ubiquitination events. Among the remaining annotated sites, 740 correspond to ubiquitination sites experimentally validated in human samples, while 5 were listed only as homology-based entries, hence, sites observed in orthologous proteins from other species but not previously confirmed in humans (Figure 4D). Our dataset therefore not only expands the catalog of known ubiquitination sites with a substantial number of novel modifications but also provides the first experimental validation of multiple conserved sites that had been inferred from cross-species evidence but never demonstrated in human proteins.

## 3. Discussion

In this study, we systematically mapped candidate substrates of the four members of the human UCH deubiquitinase family using a quantitative proteomics workflow based on DUB silencing, bioUb-mediated enrichment of ubiquitinated proteins, and label-free mass spectrometry. Our analysis revealed that UCHL1, UCHL3, UCHL5, and BAP1 regulate largely distinct subsets of ubiquitinated proteins, with limited overlap between family members.

The recovery of HIF-1α as a candidate UCHL1 substrate provides an important benchmark for our approach, as UCHL1 has previously been shown to directly deubiquitinate and stabilize HIF-1α under hypoxic conditions [41]. EP300 was also enriched in the UCHL1 dataset and, as a key coactivator of HIF-1α-dependent transcription [41], may reflect recovery of components of the same hypoxia-associated regulatory network.

Indeed, the identification of EP300, together with other large proteins such as SON and SRRM2, should be interpreted with caution. Given the relatively restricted active-site architecture of UCH family enzymes [11], it remains unclear whether these proteins represent direct deubiquitination targets of UCHL1 or are recovered indirectly through their participation in multiprotein complexes, ubiquitin-dependent signaling pathways, or broader cellular responses to UCHL1 perturbation. Interestingly, this subset of candidates was enriched in nuclear proteins, more specifically, in nuclear speckle (i.e., SON and SRRM2) and chromatin-associated proteins (i.e., EP300, CREB3L1 and CHD8). While UCHL1 is primarily cytoplasmic, accumulating evidence suggests that it can also participate in nuclear-associated processes [42]. Thus, the presence of nuclear proteins within the UCHL1 candidate list is not necessarily inconsistent with current views of UCHL1 biology, although further studies will be required to determine whether these associations reflect direct nuclear functions of UCHL1 or indirect consequences of perturbing UCHL1-regulated networks.

Besides UCHL1, the putative substrate profiles of UCHL3, UCHL5, and BAP1 were consistent with their known biological functions while also suggesting previously unrecognized connections. For instance, UCHL3 candidates comprised proteins linked to diverse roles such as cytoskeletal organization and nuclear transport, suggesting roles that extend beyond established UCHL3 functions in apoptosis and DNA repair [43]. In contrast, UCHL5 candidates were more closely associated with proteostasis, ER-associated degradation, and chromatin regulation, consistent with its well-characterized role as a proteasome-associated deubiquitinase [19]. Similarly, the substrate profile of the BAP1 was broadly aligned with its established functions in transcriptional and chromatin regulation [44], while also implicating additional pathways related to cell survival and signaling.

A major finding of this study is the pronounced substrate partitioning across the UCH family. Despite their conserved catalytic domains, each enzyme was associated with a largely distinct candidate substrate profile, suggesting that UCH specificity is influenced not only by catalytic properties but also by context-dependent mechanisms, including protein–protein interactions and regulatory constraints. These findings support the concept that structurally related DUBs can regulate distinct cellular pathways through selective substrate engagement.

Nevertheless, despite the overall partitioning of candidate substrates, several points of convergence were observed between UCH family members. For example, ATP6V0A1 was common to the UCHL1 and UCHL3 datasets and, together with dataset-specific putative substrates (RAB1 and PIK3C3, respectively), points to a potential role for both DUBs in phagocytic vesicle regulation. By contrast, GOPC was shared between UCHL1 and UCHL5, suggesting potential convergence on lysosomal and trafficking-related processes. Notably, MCL1 was identified following depletion of UCHL3, UCHL5, and BAP1 highlighting apoptosis and cell-survival regulation as a potential point of convergence among UCH family members. Together, these findings reveal largely distinct but partially overlapping candidate-substrate networks, supporting the view that UCH enzymes may regulate diverse cellular processes while sharing selected functional nodes.

Importantly, the proteins identified here should be regarded as candidate substrates rather than definitively validated direct targets. Increased ubiquitination following DUB depletion may arise through direct or indirect mechanisms. Although our analyses did not reveal evidence of a substantial global alteration in ubiquitination following UCH depletion, more subtle changes in ubiquitin availability cannot be excluded. Moreover, the absence of matched total-proteome measurements prevents us from unequivocally distinguishing changes in bioUb enrichment from changes in total protein abundance. Future studies integrating matched total-proteome and ubiquitinome measurements will be required to resolve these effects.

Additionally, our proteomics experiment resulted in the identification of 851 unique ubiquitination sites, including 106 not previously reported, thereby expanding the current catalog of experimentally identified ubiquitination sites. Although ubiquitination sites could be identified for a subset of candidate substrates, protein-level enrichment and site-level MS identification represent distinct analytical steps and therefore do not necessarily yield complete correspondence. Thus, the absence of a detected diGly site does not necessarily indicate the absence of ubiquitination, as detection of individual ubiquitinated peptides depends on site occupancy and peptide-specific properties.

Overall, our results provide a systematic resource of candidate substrates and ubiquitination sites associated with the UCH family of deubiquitinases and reveal substantial functional specialization among family members. The largely distinct yet partially overlapping candidate-substrate profiles, together with the identification of previously unreported diGly sites, provide a framework for defining the molecular mechanisms through which individual UCH enzymes contribute to the regulation of cellular signaling pathways.

## 4. Materials and Methods

### 4.1. Cell Culture and Transfection

HEK293 cells were maintained in DMEM/F-12 medium supplemented with 10% fetal bovine serum (FBS), 100 U/mL penicillin, and 100 µg/mL streptomycin (all from Gibco, Waltham, MS, USA) at 37 °C in a humidified atmosphere containing 5% CO_2_. For each experimental condition and biological replicate, 1.35 × 10^6^ cells were seeded in 150 mm culture dishes. Twenty-four hours after seeding, the culture medium was replaced with antibiotic-free medium, and cells were transfected with 10 nM siRNA using Lipofectamine RNAiMAX (Invitrogen, Waltham, MS, USA) according to the manufacturer’s instructions. Silencer Select validated siRNAs (Ambion, Waltham, MS, USA) targeting each UCH family member were used as follows: UCHL1 (s14724 and s14725), UCHL3 (s15439 and s15440), UCHL5 (s15742 and s15743), and BAP1 (s38540 and s38541). A scrambled siRNA (Ambion, #4390843) served as the negative control.

Forty-eight hours after seeding, cells were transfected with the bioUb plasmid using Lipofectamine 3000 (Invitrogen), and biotin was added to the culture medium at a final concentration of 50 µM. Cells were harvested 24 h later (72 h after seeding), pelleted by centrifugation, and stored at −80 °C until use. Unless otherwise indicated, all experiments were performed in three independent biological replicates.

### 4.2. Western Blotting

Proteins were separated on 4–12% Bolt Bis-Tris Plus SDS–PAGE gels (Invitrogen) and transferred onto PVDF membranes using the iBlot Dry Blotting System (Invitrogen). Membranes were blocked and incubated with primary antibodies against UCHL1 (mouse, Developmental Studies Hybridoma Bank (DSHB), Iowa City, IA, USA), UCHL3 (mouse, DSHB), UCHL5 (rabbit, Abcam, Cambridge, UK), BAP1 (mouse, Santa Cruz Biotechnology, Dallas, TX, USA), or tubulin (mouse, DBHS), followed by HRP-conjugated goat anti-mouse (Invitrogen, #62-6520, 1:4000) or goat anti-rabbit (#7074, Cell Signaling Technology, Danver, MA, USA) secondary antibodies. Immunoreactive bands were detected using Clarity Western ECL substrate (Bio-Rad, Hercules, CA, USA) and visualized with a ChemiDoc MP Imaging System (Bio-Rad).

### 4.3. Biotin Pull-Down

Biotin pull-down assays were performed as previously described [37]. Briefly, cell pellets were lysed in 8 M urea and 1% SDS in PBS supplemented with 50 mM N-ethylmaleimide (NEM; Sigma, Chicago, IL, USA) and a protease inhibitor cocktail (Roche, Basel, Switzerland). Lysates were homogenized by passing them ten times through a 20-gauge needle and clarified by centrifugation at 14,000× *g* for 5 min at 4 °C. Supernatants were desalted using PD-10 columns (GE Healthcare, Chicago, IL, USA) equilibrated in binding buffer (3 M urea, 1 M NaCl, 0.25% SDS, 50 mM NEM in PBS).

Desalted lysates were incubated with 150 µL NeutrAvidin agarose resin (Thermo Scientific, Waltham, MA, USA) for 40 min at room temperature followed by 2 h 20 min at 4 °C. Beads were sequentially washed with WB1 (8 M urea, 0.25% SDS in PBS), WB2 (6 M guanidine-HCl), WB3 (6.4 M urea, 1 M NaCl, 0.2% SDS in PBS), WB4 (4 M urea, 1 M NaCl, 10% isopropanol, 10% ethanol, 0.2% SDS in PBS), WB1, lysis buffer, and WB5 (2% SDS in PBS). Bound ubiquitinated proteins were eluted at 95 °C for 5 min in elution buffer (250 mM Tris-HCl, pH 7.5, 40% glycerol, 4% SDS, 0.2% bromophenol blue, and 100 mM DTT). Resin was removed using 0.8 µm PES centrifugal filter units (Sartorius, Göttingen, Germany).

### 4.4. In-Gel Digestion and Peptide Extraction

Eluted proteins were separated on 4–12% Bolt Bis-Tris Plus gels (Invitrogen) and stained with GelCode Blue (Thermo Scientific). After removal of avidin-derived bands, each lane was divided into four gel slices and subjected to in-gel tryptic digestion as previously described [45]. Briefly, proteins were reduced with DTT, alkylated with chloroacetamide, and digested overnight at 37 °C with sequencing-grade trypsin. Peptides were extracted using acetonitrile, dried by vacuum centrifugation, and stored at −20 °C until LC-MS/MS analysis.

### 4.5. LC-MS/MS Analysis

Peptide samples were analyzed at the Proteomics Core Facility SGIKER (University of the Basque Country, EHU) using an EASY-nLC 1000 nanoLC system coupled to a Q Exactive mass spectrometer (Thermo Scientific). Peptides were first loaded onto an Acclaim PepMap 100 precolumn and subsequently separated on an Acclaim PepMap RSLC analytical column using a 45 min linear gradient from 2% to 40% acetonitrile in 0.1% formic acid at a flow rate of 300 nL/min.

Full MS spectra were acquired over an *m*/*z* range of 300–1850 at a resolution of 70,000 (at m/z 200), followed by data-dependent acquisition of the ten most intense precursor ions using higher-energy collisional dissociation (HCD; normalized collision energy of 28). MS/MS spectra were acquired at a resolution of 17,500. Automatic gain control (AGC) targets were set to 3 × 10^6^ for MS scans and 5 × 10^5^ for MS/MS scans, with a maximum injection time of 120 ms. Dynamic exclusion was set to 45 s, and singly charged or unassigned precursor ions were excluded from fragmentation. Data acquisition was performed using Xcalibur software version 4.1.

### 4.6. Data Processing and Bioinformatic Analysis

Raw MS files were processed using MaxQuant (version 1.5.3.17). MS/MS spectra were searched against the Homo sapiens UniProt reference proteome using the integrated Andromeda search engine. Carbamidomethylation of cysteine was specified as a fixed modification, whereas methionine oxidation, protein N-terminal acetylation, and Lys-GlyGly modification were included as variable modifications. Trypsin specificity was selected, allowing cleavage before proline or Asp-Pro, with a maximum of two missed cleavages. Precursor and fragment ion mass tolerances were set to 8 ppm and 20 ppm, respectively. Peptide-spectrum matches and proteins were filtered at a false discovery rate (FDR) of 1%. The match-between-runs option was enabled using a 1.5 min matching window and a 20 min alignment window. Label-free quantification was performed using the MaxLFQ algorithm.

Protein groups identified only by site, reverse hits, contaminants, proteins lacking unique peptides, or proteins without quantitative values were excluded using Perseus (version 1.5.6.0). LFQ intensities were used for protein quantification, whereas raw GlyGly peptide intensities were normalized by median subtraction before statistical analysis. Missing values were imputed from a normal distribution (width = 0.3; downshift = 1.8) to simulate low-abundance signals below the detection limit. Statistical significance was assessed using two-tailed Student’s *t*-tests. Proteins exhibiting a fold change greater than 2 and a *p* value < 0.05 were considered significantly regulated. Candidate DUB substrates were defined as significantly enriched proteins with no imputed values in any of the three biological replicates of at least one experimental condition or with a maximum of one imputed value per condition.

## Figures and Tables

**Table 1:** The most compelling candidate substrates of UCH family of DUBs. Proteins were selected based on their enrichment (log_2_ ratio > 1 & *p*-value < 0.05) in the siDUB/siCtr comparison. “Shared with” column indicates candidate substrates identified for more than one UCH DUB. diGly sites column lists the lysine residues bearing diGly modifications identified by mass spectrometry.

	UniProt ID	Gene	Peptides (Unique)	log_2_ Ratio (siDUB/siCtr)	*p*-Value	Shared with	diGly Sites
UCHL1	**Q93050-1**	**ATP6V0A1**	**5 (3)**	**2.20**	**0.00073**	**UCHL3**	
	**Q9HD26-2**	**GOPC**	**5 (5)**	**1.22**	**0.00083**	**UCHL5**	
	P61313	RPL15	6 (6)	1.06	0.00093		K83, K140
	P30279	CCND2	3 (3)	2.83	0.00170		
	P06241	FYN	8 (2)	1.07	0.00177		
	Q5SW79-2	CEP170	15 (15)	1.05	0.00251		
	Q49A88-4	CCDC14	3 (3)	2.49	0.00396		
	P18583-10	SON	13 (13)	1.17	0.00542		
	Q15833-2	STXBP2	6 (6)	2.23	0.00567		
	Q9Y6N7-6	ROBO1	15 (15)	1.95	0.00714		
	Q9BTC8-2	MTA3	8 (4)	1.58	0.00757		
	Q09472	EP300	9 (5)	1.02	0.01176		
	P61026	RAB10	4 (3)	1.20	0.02088		
	Q16665-2	HIF-1α *	5 (5)	1.04	0.02437		
	Q96BA8-2	CREB3L1	2 (2)	1.58	0.02678		
	Q96Q15-3	SMG1	7 (7)	1.57	0.02824		
	P38936	CDKN1A	4 (4)	1.23	0.02834		
	Q12923-3	PTPN13	5 (5)	2.17	0.03321		
	Q9Y5A9	YTHDF2	5 (2)	1.13	0.03418		
	Q9UQ35	SRRM2	5 (5)	1.04	0.03643		
	P02810	PRH1	2 (2)	1.72	0.03995		
	Q9HCK8	CHD8	6 (5)	1.19	0.04754		
UCHL3	Q9Y6W5	WASF2	2 (2)	2.28	0.00601		
	**Q07820**	**MCL1**	**4 (4)**	**2.01**	**0.01609**	**UCHL5, BAP1**	
	Q9Y605	MRFAP1	4 (4)	1.73	0.01306		
	**Q93050-1**	**ATP6V0A1**	**5 (3)**	**1.66**	**0.01026**	**UCHL1**	
	Q06546	GABPA	3 (3)	1.42	0.04054		
	Q9UKX7-2	NUP50	5 (5)	1.31	0.00688		
	P49757-8	NUMB	1 (1)	1.18	0.02268		
	Q8NEB9	PIK3C3	1 (1)	1.02	0.04889		
UCHL5	P01112	HRAS	2 (1)	3.16	0.00494		
	Q9BQE4	VIMP	3 (3)	3.03	0.00005		
	Q8TF66	LRRC15	5 (5)	2.98	0.00607		
	Q9HC35-2	EML4	5 (5)	2.42	0.01169		
	**Q9HD26-3**	**GOPC**	**3 (3)**	**2.35**	**0.00011**	**UCHL1**	
	O75822-3	EIF3J	4 (4)	2.31	0.00036		
	P04745	AMY1A	13 (13)	2.22	0.00146		
	Q13685	AAMP	2 (2)	2.06	0.03097		
	**Q07820**	**MCL1**	**6 (6)**	**1.95**	**0.00005**	**UCHL3, BAP1**	K194, K197
	Q96GF1	RNF185	1 (1)	1.65	0.01790		
	Q9NRV9	HEBP1	1 (1)	1.57	0.01744		
	Q9Y4A5-2	TRRAP	7 (7)	1.56	0.01554		
	Q9Y4B4	RAD54L2	11 (11)	1.55	0.00198		
	Q13206	DDX10	10 (10)	1.29	0.00061		
	Q8N302	AGGF1	3 (3)	1.27	0.00038		
	Q96DA0	ZG16B	4 (4)	1.23	0.01634		
	P12273	PIP	9 (9)	1.16	0.00473		
	Q9BW83-2	IFT27	2 (2)	1.15	0.02335		
	P63172	DYNLT1	1 (1)	1.03	0.00063		
BAP1	Q9UBN6	TNFRSF10D	2 (2)	3.40	0.00363		
	Q13769	THOC5	2 (2)	2.86	0.00058		
	Q9Y6W3	CAPN7	3 (3)	2.24	0.00065		
	**Q07820**	**MCL1**	**3 (3)**	**2.07**	**0.04690**	**UCHL3, UCHL5**	
	P08581	MET	4 (4)	1.92	0.04195		
	Q9NVF7	FBXO28	4 (4)	1.25	0.00883		
	O00303	EIF3F	8 (8)	1.21	0.00035		
	Q8N0X7	SPG20	22 (22)	1.19	0.00036		K62, K70, K362, K465
	Q58EX2-3	SDK2	3 (3)	1.06	0.02067		
	P22314-2	UBA1	48 (48)	1.03	0.00218		K595

* Known substrates of the indicated DUB. In bold, putative substrates shared by several UCH family members.

## Data Availability

The mass spectrometry proteomics data have been deposited with the ProteomeXchange Consortium via the PRIDE partner repository with the dataset identifier PXD081376.

## Supplementary Materials

The following supporting information can be downloaded at: https://www.mdpi.com/article/10.3390/ijms27209217/s1.

## Abbreviations

The following abbreviations are used in this manuscript:

BAP1	BRCA1-associated protein 1
DUB	Deubiquitinase
HIF-1α	Hypoxia-inducible factor 1α
JAMM	JAB1/MPN/MOV34
LFQ	Label-free quantification
MINDY	MIU-containing novel DUB
MJD	Machado–Josephin domain-containing proteases
MS	Mass spectrometry
NEDD8	Neural Precursor Cell Expressed, Developmentally Down-Regulated 8
OTU	Ovarian tumor proteases
SUMO	Small ubiquitin-like modifier
UCH	Ubiquitin C-terminal hydrolase
ULD	UCHL37-like domain
USP	Ubiquitin-specific proteases
ZUP1	zinc finger-containing ubiquitin peptidase 1
