# Supplementary material for: Systematic Identification of Candidate Substrates of the UCH Family of Deubiquitinases
Source: Int J Mol Sci. 2026 Oct 17;27(20):9217. doi: 10.3390/ijms27209217 (PMC13705248; doi:10.3390/ijms27209217)
Supplement: Supplementary file 1 [file ijms-27-09217-s001.zip › Figure S1.pdf]

Figure S1

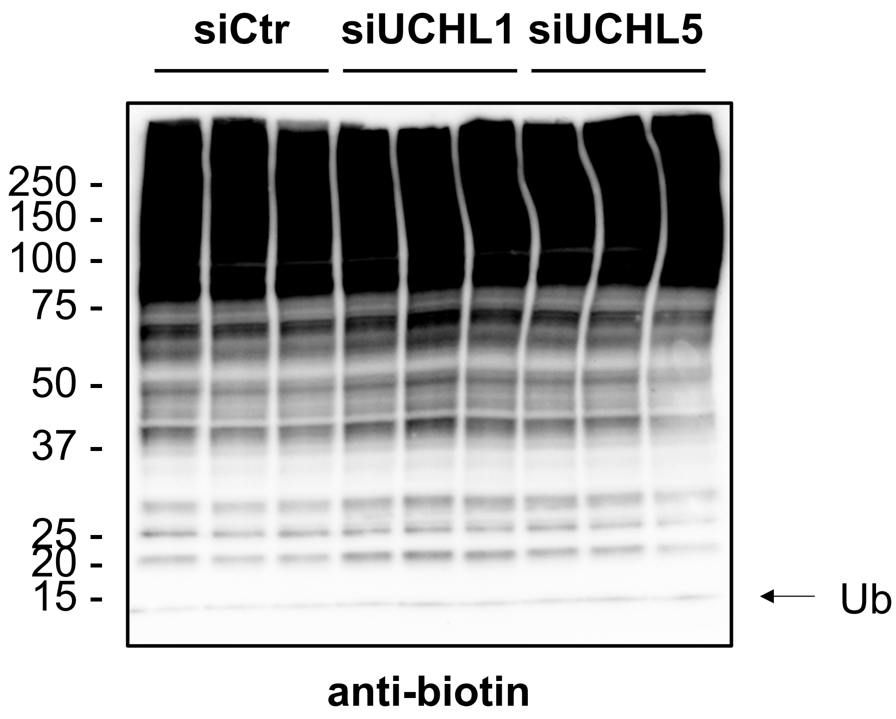
siCtr
siUCHL1
siUCHL5
250 -
150 -
100 -
75 -
50 -
37 -
25 -
20 -
15 -
Ub
anti-biotin

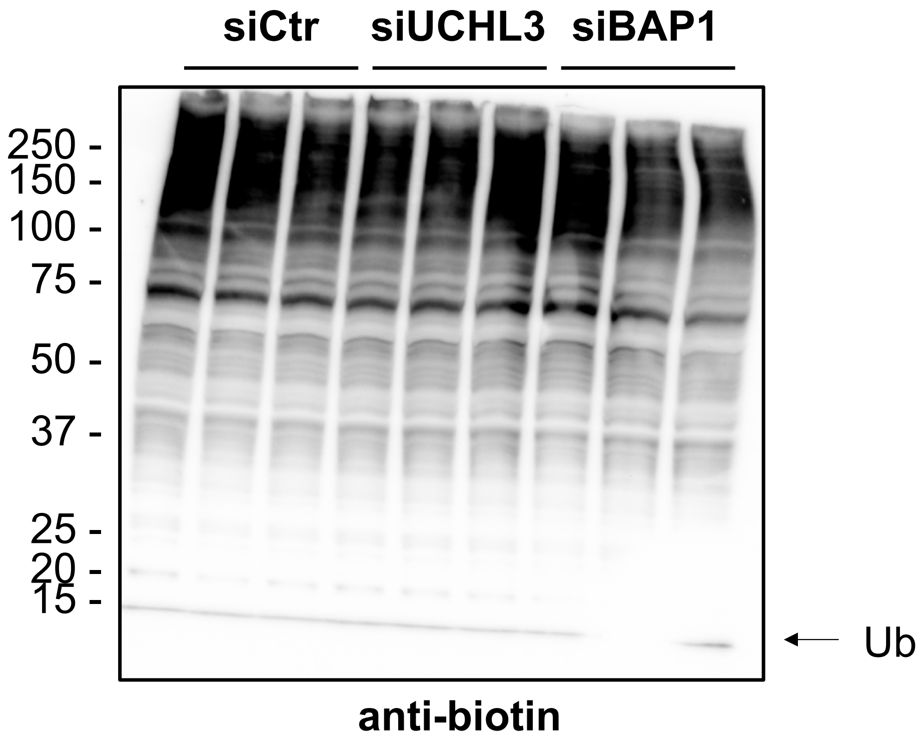
siCtr
siUCHL3
siBAP1
250 -
150 -
100 -
75 -
50 -
37 -
25 -
20 -
15 -
Ub
anti-biotin
