# Supplementary material for: Systematic Identification of Candidate Substrates of the UCH Family of Deubiquitinases
Source: Int J Mol Sci. 2026 Oct 17;27(20):9217. doi: 10.3390/ijms27209217 (PMC13705248; doi:10.3390/ijms27209217)
Supplement: Supplementary file 1 [file ijms-27-09217-s001.zip › Figure S2.pdf]

Figure S2

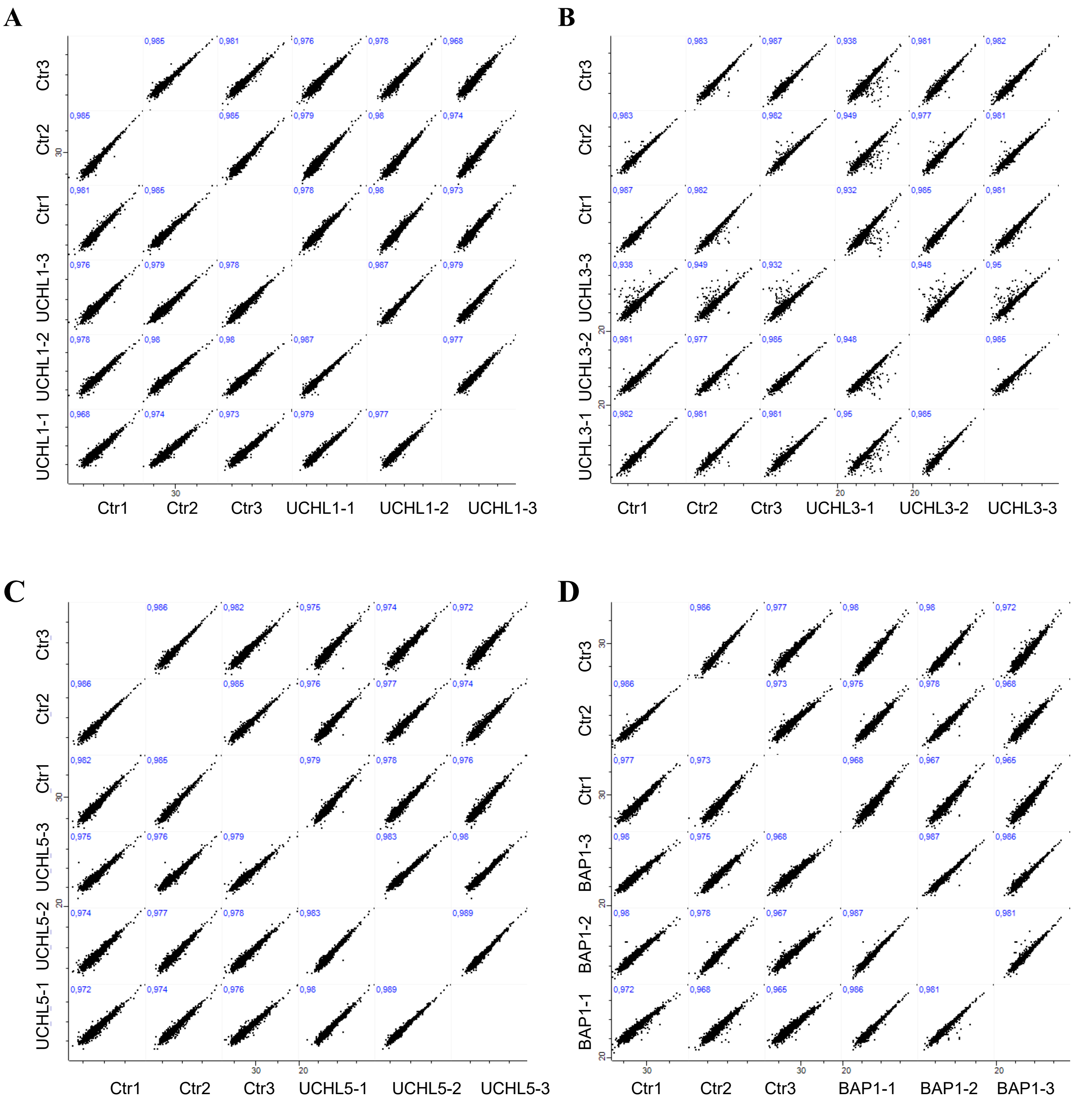
A
Ctr3
0,985 0,981 0,976 0,978 0,968
Ctr2
0,985 0,985 0,979 0,98 0,974
Ctr1
0,981 0,985 0,978 0,98 0,973
UCHL1-3
0,976 0,979 0,978 0,987 0,979
UCHL1-2
0,978 0,98 0,98 0,987 0,977
UCHL1-1
0,968 0,974 0,973 0,979 0,977
Ctr1 Ctr2 Ctr3 UCHL1-1 UCHL1-2 UCHL1-3
B
Ctr3
0,983 0,987 0,938 0,981 0,982
Ctr2
0,983 0,982 0,949 0,977 0,981
Ctr1
0,987 0,982 0,932 0,985 0,981
UCHL3-3
0,938 0,949 0,932 0,948 0,95
UCHL3-2
0,981 0,977 0,985 0,948 0,985
UCHL3-1
0,982 0,981 0,981 0,95 0,985
Ctr1 Ctr2 Ctr3 UCHL3-1 UCHL3-2 UCHL3-3
C
Ctr3
0,986 0,982 0,975 0,974 0,972
Ctr2
0,986 0,985 0,976 0,977 0,974
Ctr1
0,982 0,985 0,979 0,978 0,976
UCHL5-3
0,975 0,976 0,979 0,983 0,98
UCHL5-2
0,974 0,977 0,978 0,983 0,989
UCHL5-1
0,972 0,974 0,976 0,98 0,989
Ctr1 Ctr2 Ctr3 UCHL5-1 UCHL5-2 UCHL5-3
D
Ctr3
0,986 0,977 0,98 0,98 0,972
Ctr2
0,986 0,973 0,975 0,978 0,968
Ctr1
0,977 0,973 0,968 0,967 0,965
BAP1-3
0,98 0,975 0,968 0,987 0,986
BAP1-2
0,98 0,978 0,967 0,987 0,981
BAP1-1
0,972 0,968 0,965 0,986 0,981
Ctr1 Ctr2 Ctr3 BAP1-1 BAP1-2 BAP1-3
